# Epidemiological and Genetic Characteristics of Rabies Virus Transmitted Through Organ Transplantation

**DOI:** 10.3389/fcimb.2018.00086

**Published:** 2018-03-27

**Authors:** Jingfang Chen, Guang Liu, Tao Jin, Rusheng Zhang, Xinhua Ou, Heng Zhang, Peng Lin, Dong Yao, Shuilian Chen, Meiling Luo, Fan Yang, Dana Huang, Biancheng Sun, Renli Zhang

**Affiliations:** ^1^Changsha Center for Disease Control and Prevention, Changsha, China; ^2^Shenzhen Center for Disease Control and Prevention, Shenzhen, China; ^3^China National Genebank-Shenzhen, Shenzhen, China; ^4^Infection Omics Research Institute, BGI-Shenzhen, Shenzhen, China; ^5^BGI Education Center, University of Chinese Academy of Sciences, Shenzhen, China

**Keywords:** rabies, organ transplantation, next-generation sequencing, phylogenetic analysis, transmission

## Abstract

In January 2016, two patients died of rabies after receiving kidney transplants from a common organ donor at a hospital in Changsha, Hunan, China. The medical records, epidemiological data of the organ donor, two kidney and a liver recipients were reviewed. Intravitam saliva samples of the two kidney recipients were tested for rabies virus (RABV) using real-time RT-PCR, and the nucleoprotein (*N*) gene was amplified and sequenced by Sanger sequencing. Whole genome sequences were analyzed using next-generation sequencing. The *N* genes of the two kidney recipients showed 100% nucleic acid identity. Phylogenetic analysis of the complete genome, *N* and glycoprotein (*G*) genes indicated that the RABV was homologous with dog isolates from the Hunan province and belong to the China I lineage, which is widespread in China. The organ donor was a 22-month-old boy who died from unknown acute progressive encephalitis. After undergoing sub-hypothermia hibernation therapy, rabies-associated symptoms were atypical, and rabies was neglected because serum RABV-specific antibodies were negative. An unknown wound on the forehead of the donor was found 2 months before the onset of symptoms. Based on the clinical, epidemiological, and molecular findings, we speculated that the RABV initially originated in the donor from a dog bite, and was then transmitted to the recipients by organ transplantation. An uncertain exposure history and misdiagnosis played important roles in the spread of the RABV. Rabies should be considered in patients with acute progressive encephalitis of unexplained etiology, especially in potential organ donors.

## Introduction

Rabies is a zoonotic disease caused by the rabies virus (RABV) that belongs to the Lyssavirus genus within the family Rhabdoviridae. RABV is a single-stranded, negative-sense, non-segmented RNA virus with a 12,000 nucleotide genome. Rabies is an acute infectious disease associated with almost 100% mortality in unvaccinated hosts. Globally, 60,000 people die of rabies annually, with Asia accounting for ~80% of deaths (Knobel et al., 2005; Wunner and Briggs, 2010). Rabies is a serious public health problem in China, which reported the second highest number of human rabies cases with the epidemic area almost expanding across the entire country (Zhang et al., 2009). In China, domestic dogs act as the main viral reservoir and are primarily responsible for the dissemination of the disease, and almost 95% of human cases have been associated with dog bites (Tang et al., 2005; Zhang et al., 2011).

Organ transplantation is considered the therapy of choice for end-stage organ failure. Unexpected transmission of infection from donors to recipients is infrequent and have been reported before, including bacterial, fungal, viral, and protozoal infections (Baddley et al., 2010; Fishman et al., 2012; Giani et al., 2014). Besides some common pathogens, such as hepatitis B virus, hepatitis C virus, human immunodeficiency virus, other rare and unusual viral infection through organ transplantation like RABV, West Nile virus have been observed and resulted in highly visible recipients death (Iwamoto et al., 2003; Fishman and Grossi, 2014). Since the first report of rabies transmission by corneal transplantation in 1979 (Houff et al., 1979), few cases ascribed to solid organ or vascular-tissue transplantation have been reported (Srinivasan et al., 2005; Vora et al., 2013). A transplantation-infectious case was reported in China in 2015 (Zhou et al., 2016). In January 2016, two kidney transplant recipients, who received organs from the same donor, presented with vomiting, abdominal pain, and right lower extremity weakness. The recipients subsequently developed excessive salivation and altered mental status. Real-time reverse-transcriptase polymerase chain reaction (RT-PCR) on saliva specimens detected RABV nucleic acids. The rabies diagnosis was confirmed by the Hunan Center for Disease Control and Prevention (CDC) and China CDC. To determine whether the RABV infection was donor-derived and the possible infectious source, clinical and epidemical data of the organ donor and all recipients were collected and whole genome analysis of the virus from the recipients were conducted by next generation sequencing (NGS).

## Materials and methods

### Clinical and epidemiological data collection

The medical records of the organ donor and infected transplant recipients were reviewed to characterize the clinical courses and diagnostic evaluations. Interviews with family members of the deceased organ donor and recipients were conducted to track the possible sources of infection.

### Specimen collection and RABV identification

The antemortem rabies diagnoses for the two deceased kidney recipients were performed using saliva specimens. Our study was carried out in accordance with the recommendations of ethical guidelines of human biomedical research and Changsha CDC ethical committee with written informed consent from all subjects. All subjects gave written informed consent in accordance with the Declaration of Helsinki. The protocol was approved by the ethical committee of Changsha CDC.

Viral RNA was extracted using the QIAamp Viral RNA Mini Kit (Qiagen, Hilden, Germany) according to the manufacturer's instructions. RABV identification was conducted using real-time RT-PCR in Changsha CDC using a commercial detection kit (Jiangsu bioperfectus technologies, Taizhou, China). Special primers N55 (forward: 5′-ATGTAACACCTCTACAATGG-3′) and N1586 (reverse: 5′-CAGTCTCYTCNGCCATCT-3′) were synthesized (TAKARA, Dalian, China) and used to amplify the full length of the *N* gene (Chiou et al., 2014), and the PCR products were used for Sanger sequencing.

### Viral cDNA synthesis and NGS

Reverse transcription was conducted using Superscript II reverse transcriptase (Invitrogen, Carlsbad, CA, USA). Viral cDNA were obtained using random hexamer primers. The sequencing libraries were prepared with an insert size of 160 bp according to the manufacturer's instructions on the Illumina Hiseq platform. The libraries were then sequenced by 100-bp paired-end sequencing using a HiSeq 4000 Sequencer.

### Sequencing data analysis

The raw NGS reads were processed by filtering out the low-quality reads (20% bases with qualities <Q20), any duplication, poly-Ns (with 8Ns), and host contaminated reads (SOAPaligner <5 mismatches) as published previously (Li et al., 2008, 2009). The remaining high-quality reads were mapped to RABV reference sequences downloaded from GenBank (NCBI) to choose the best match reference. Reference-based assembly was performed using MAQ (version 0.7.1). Denovo assembly, to correct reference assembly errors such as inserts and mismatches, was executed using SOAP denovo2 software (version 2.04; Luo et al., 2012). A second round reference-based assembly was conducted to create the final assembled sequences based on the improved sequences generated from the combination of the above two methods. Complete assembled genomes were exported in FASTA format for subsequent analysis.

### Sequence alignment and phylogenetic analysis

Sequences homology comparisons were performed using BLAST (NCBI). Worldwide 111 complete street RABV genomes, including 41 sequences from China, which represent the major Chinese phylogenetic groups, and 4 vaccine strains were downloaded from GenBank (NCBI). Additionally, 89 complete Chinese sequences of the *N* gene and 112 *G* gene sequences were retrieved for further analysis. Multiple sequence alignments were performed using the Clustal W (Version 2.1) program and phylogenetic trees were constructed using the maximum-likelihood method with bootstrap analysis (*n* = 1,000), using MEGA version 5.1 (MEGA).

## Results

### Clinical reviews of the organ donor and recipients

#### Organ donor

The organ donor was a 22-month-old male who lived with his grandparents in a village in Yuanjiang City, Hunan province. On Nov 25, 2015, the boy presented with a fever (40.3°C) and anxiety, and refused to eat, drink, or sleep. His symptoms did not resolve after the administration of antifebrile drugs at the local county hospital.

On Dec 2, 2015, he was admitted to a provincial hospital with fever and vomiting. He appeared fearful and hyperspasmic. Physical and laboratory examinations were as follows: temperature, 38.5°C; pulse, 189/min; respirations, 32/min; blood pressure, 101/68 mm Hg; white blood cell count, 21.62 × 10^9^/L (67% neutrophils; 28.2% lymphocytes); serum K^+^ <2 mM. Chest radiographs and head computed tomography scans were normal. Sub-hypothermia hibernation therapy was administered within 48 h of admission, and his vital signs became stable.

On Dec 8, 2015, the child became unresponsive, but his eyes remained open. Viral encephalitis was suspected. Hand, foot and mouth disease, rabies, and food poisoning were considered in the differential diagnosis. The following day, his condition deteriorated rapidly, and he was transferred to another provincial children hospital. Tracheal aspirate antibody tests for influenza virus, parainfluenza virus, respiratory syncytial virus, and adenovirus were negative. Serum antibody tests for rabies immunoglobulin were negative. On Dec 10, 2015, the boy was declared brain-dead and donor eligibility screening and testing, in accordance with the organ donation law in China, did not reveal any contraindications to transplantation. Therefore, the donor's kidneys and liver were removed for transplantation.

#### Kidney recipient 1

**Figure 1:**
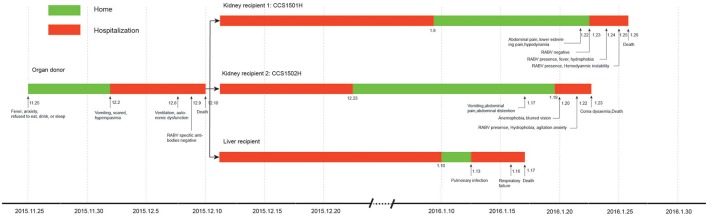
Clinical course of the transplant donor and 3 recipients in a rabies outbreak associated with solid organ transplantation.

Kidney recipient 1 was a 47-year-old woman, on Jan 23, 2016, 44 days post-transplantation, she was readmitted to the hospital complaining of lower abdominal and lower extremity pain, and hypodynamia. A saliva sample was collected and sent to Changsha CDC for RABV nucleic acid test because another kidney recipient from the same organ donor had been diagnosed with rabies by Changsha CDC, and the result was negative (Figure 1). The following day she developed fever and anxiety and refused to drink water. Real-time RT-PCR on salvia specimens revealed the presence of RABV. She subsequently developed excessive salivation, hemodynamic instability, and coma, and died on Jan 26, 2016.

#### Kidney recipient 2

Kidney recipient 2 was a 29-year-old female. On 19 Jan 2016, 40 days post-transplantation, she presented to the hospital with a 2-day history of vomiting, abdominal pain, and abdominal distention. The following day, she complained of anemophobia and blurred vision. On Jan 22, 2016, she appeared fearful and agitated and refused to drink water. Real-time RT-PCR on salvia specimens revealed the presence of RABV. Her condition deteriorated rapidly eliciting a decrease in blood pressure and loss of consciousness resulting in death on Jan 23, 2016.

#### Liver recipient

The liver recipient was a 10-month-old infant who underwent transplantation at a hospital in Shanghai, China. She was readmitted on Jan 13, 2016, due to a pulmonary infection and died on Jan 17, 2016, from respiratory failure.

#### Epidemiological investigation

Interviews with family members revealed that the donor had an unclear history of an animal bite. His grandmother recalled that 2 months before his symptoms onset she had heard the boy crying outdoors and found an unexplained wound on his forehead. They had no pets, the boy had no travel history, and he had not been vaccinated for rabies. Epidemiological investigations revealed that many stray dogs roamed in the donor's village. Interviews with the two kidney recipients and their families revealed no animal exposure history or rabies prophylaxis. Neither recipient had a history of chronic disease, blood-borne infection, trauma, or blood transfusion.

#### Summary of sequencing data of the RABV genome

The complete coding regions of the *N* genes were obtained using specific primers and Sanger sequencing from the saliva specimens designated as CCS1501H and CCS1502H, respectively. The *N* gene sequences of these two patients were 100% identical and showed 99.9% (1348/1353) homology with strain CHN0903D/Hunan/dog/2009 which was isolated from a dog brain in Hunan, China.

High-throughput NGS was performed to obtain the complete genomes of the viruses in the recipients. A total of 31,047,583 paired-end filtered reads were obtained for the two samples after removing low-quality and contaminated reads. However, only 0.02–0.03% of the filtered reads could be mapped to the viral sequences download from a public database (Table 1).

**Table 1:** Summary of the two kidney recipients NGS data.

**Sample**	**Total reads**	**Filtered reads**	Virus reads^*^	**RABV reads**
CCS1501H	43,599,134	17,510,082	5,699 (0.03%)	772
CCS1502H	43,584,854	13,537,501	2,465 (0.02%)	37

* *The percentage of virus reads compared to filtered reads*.

For sample CCS1501H, the complete RABV genome including the five coding regions was obtained. However, only the full-length *N* gene was obtained in sample CCS1502H, and the other four coding regions were missing. Only a saliva sample was collected because the condition of this recipient deteriorated very rapidly and died on the next day. The viral RNA amount was too small to obtain a good sequencing libraries and the complete genome assembly was failure.

The *N* genes between the NGS and the Sanger sequencing were compared using BLAST to evaluate the accuracy of the reference-based assembly, which was 100%. The *N* gene sequences of the two strains and complete genome of CCS1501H have been deposited in GenBank (accession numbers: MF459637, MF459638, and MF476106, respectively).

#### Phylogenetic analysis

**Figure 2:**
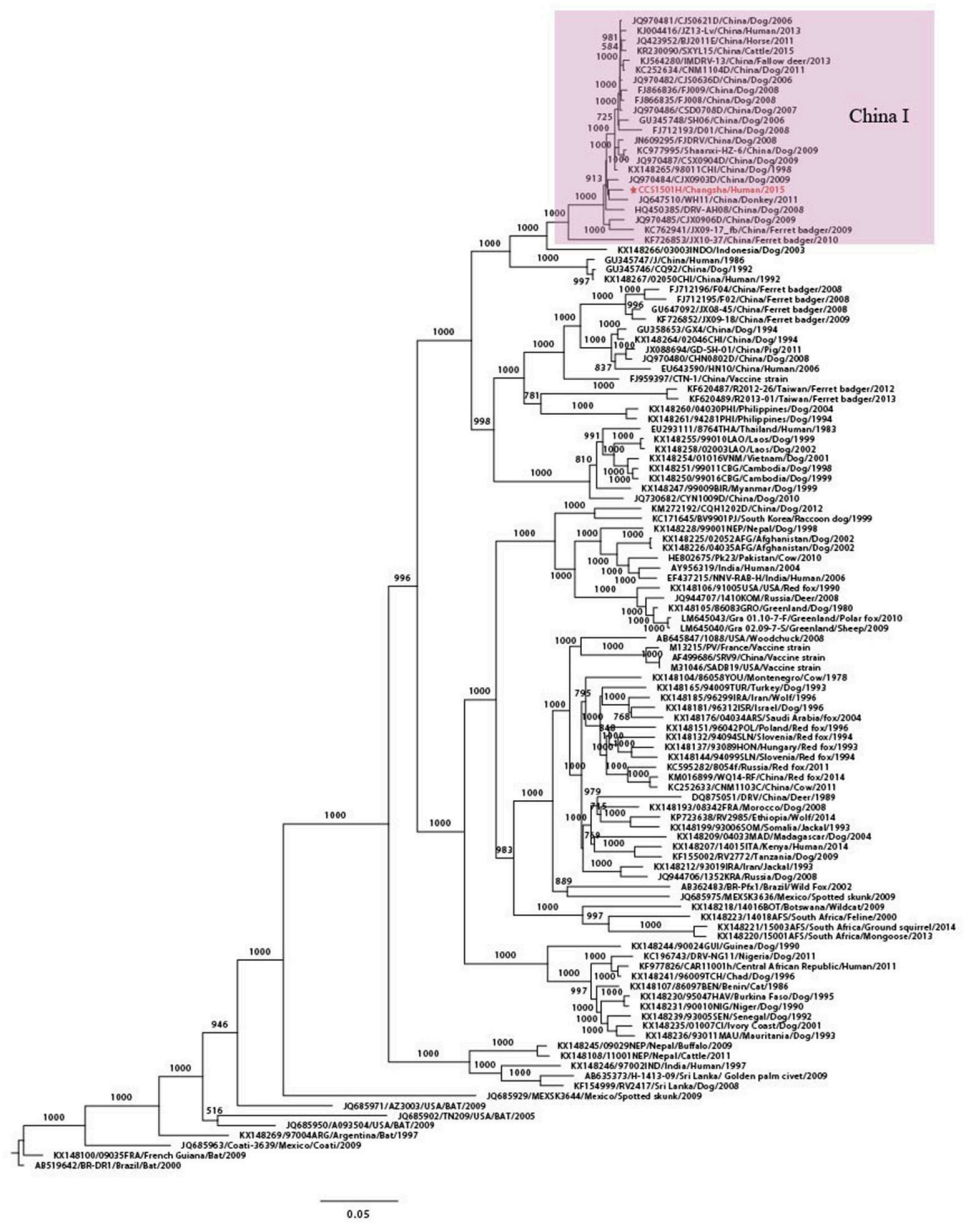
Phylogenetic tree for the genome sequence of RABV. Maximum likelihood phylogenetic tree using complete genome sequences from 116 worldwide RABV isolates. CCS1501H was marked in red. The background information for all sequences were provided in Supplementary Table S1.

Maximum likelihood phylogenetic trees were constructed, using sequences retrieved from GenBank, to improve understanding of the molecular evolution of the RABV and to determine the possible origin of RABV in the kidney recipients. Complete genome phylogenetic analysis demonstrated that, worldwide, RABVs were clustered according to geographical location and that CCS1501H was the strain closest to CJX0903D/Jiangxi/ Dog/2009 and belonged to the China I lineage that circulates mainly in dogs in China (Figure 2).

Homological analysis demonstrated that the complete genome of CCS1501H showed 99% homology with that strain. Furthermore, the five internal genes had highest nucleotide identity with RABV isolated from dogs in China. For the *N* gene, the virus shared 99% nucleotide identity with the CHN0903D/Hunan/Dog/2009 strain. For the matrix (*M*) and phosphoprotein (*P*) genes, the virus shared highest nucleotide identity (99%) with the CHN0532D/Hunan/Dog/2005 strain. For the *G* gene, the virus shared highest nucleotide identity (99%) with the CQFJ02/Chongqing/Dog/2007 strain. For the virion-associated RNA polymerase (*L*) gene, the virus shared highest nucleotide identity (99%) with the CJX0903D/Jiangxi/Dog/2009 strain (Table 2). There are a limited number of RABV sequences from the Hunan province available in the public database. Therefore, homological strains were isolated from other regions, but they all belonged to the China I lineage (Guo et al., 2013).

**Table 2:** Homology analysis of five genes from sample CCS1501H with other strains in China.

**Gene**	**Name/Region/Host/Year/Clade**	**Accession No**.	**Idenetity (%)**	**References**
*N*	CHN0903D/Hunan/Dog/2009/ChinaI	JN974848	99	This study
*P*	CHN0532D/Hunan/Dog/2005/ChinaI	EU004774	99	Guo et al., 2013
*M*	CHN0532D/Hunan/Dog/2005/ChinaI	EU004736	99	Guo et al., 2013
*G*	CQFJ02/Chongqing/Dog/2007/ChinaI	GU186388	99	This study
*L*	CJX0903D/Jiangxi/Dog/2007/ChinaI	JQ970484	99	This study

**Figure 3:**
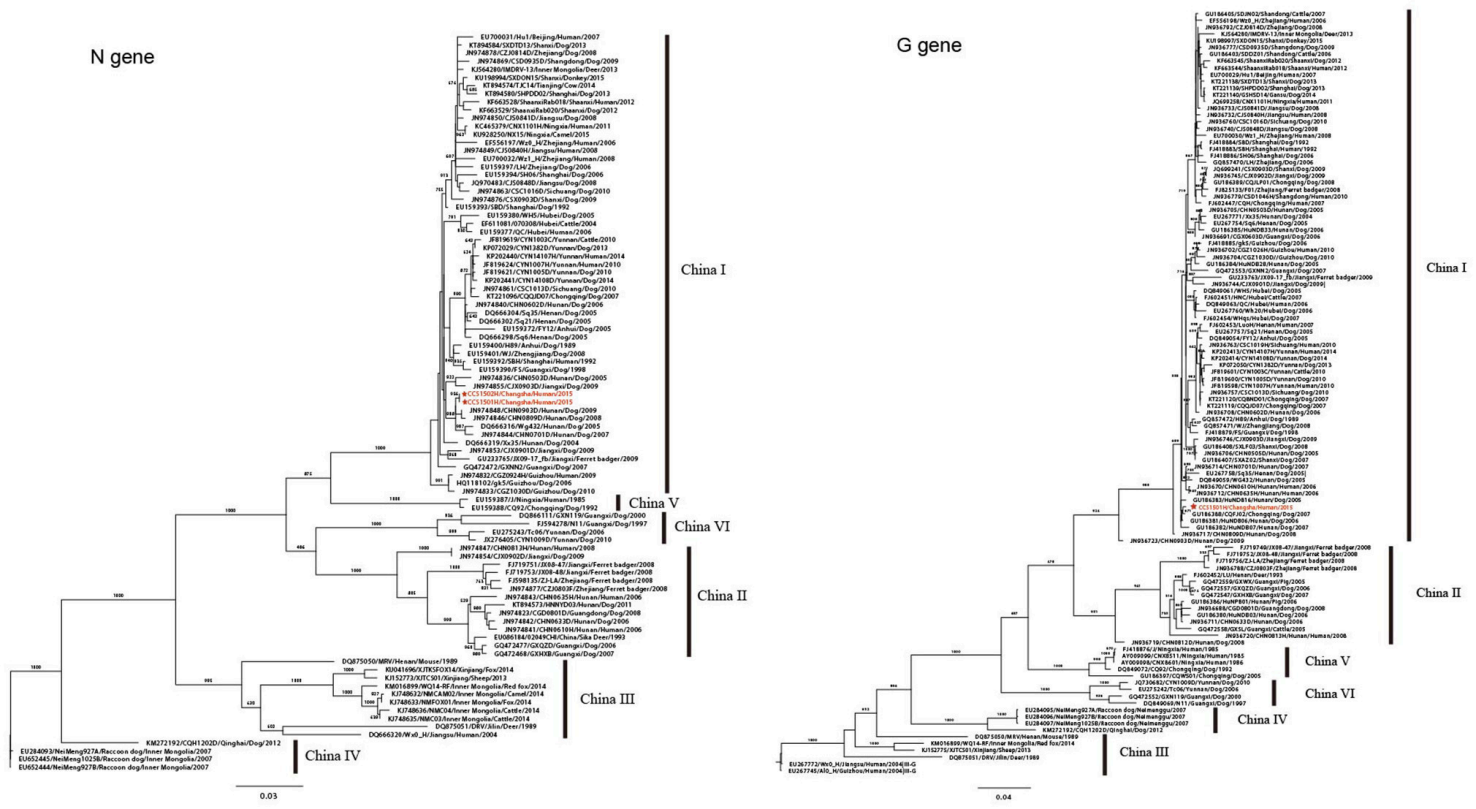
Phylogenetic trees for the *N* and *G* genes. Phylogenetic trees of 91 full-length *N* gene sequences and 113 full-length *G* gene sequences of Chinese RABV isolates. Six distinct lineages (China I–VI) are predicted with high posterior value support with the majority of isolates clustered in China I. CCS1501H and CCS1502H are marked in red. The background information for all sequences were provided in Supplementary Table S2.

To further explore the detailed phylogeographical origin of RABV, 89 complete Chinese *N* gene sequences and 112 complete Chinese *G* gene sequences were retrieved from GenBank. These strains were isolated from dog, human, deer, mouse, cattle, pig, sheep, fox, camel, and ferret badger, and circulated around 25 Chinese provinces between 1985 and 2015, which represents the major lineages of the Chinese strains, China I–VI (Guo et al., 2013; Tao et al., 2013). From the phylogenetic trees of the *N* genes of the two recipients, the virus belonged to China I lineage and was clustered with a strain isolated from a dog in Hunan in 2009 (CHN0903D/Hunan /Dog/2009; Figure 3). The *G* gene was also clustered in the China I lineage, which was closely related to the strains isolated from dogs in Hunan (HuNDB06/Hunan/Dog /2006) and Chongqing (CQFJ02/Chongqing/Dog/2007; Figure 3). These findings indicated that the RABV in the two kidney recipients might have originated from a dog in the Hunan province.

## Discussion

Rabies is a serious public health disease in China with more than 11,7500 recorded deaths and three major epidemics since 1950 (Tao et al., 2009). In China, most human rabies cases can be ascribed to dog bites, and transmission via organ transplantation is rare (Tang et al., 2005). To our knowledge, a single rabies case via organ transplantation was reported in 2015 in Beijing (Zhou et al., 2016). In this study, RABV was identified in the saliva specimens from two kidney recipients at a hospital in Hunan, Changsha, using real time RT-PCR and NGS.

Homology analysis showed that the *N* genes of the viruses from the kidney recipients shared 100% nucleic acid identity, indicating that the both viruses probably originated from the same source. The whole genome phylogenetic tree of CCS1501H demonstrated that the virus belonged to the China I lineage, which is a dog-associated, dominant lineage in the present Chinese rabies epidemic (Zhu et al., 2015). The same results were deduced from the homology comparisons and phylogenetic analysis of the *N* and *G* genes. Further detailed phylogeographical analysis showed that the virus were homologous with dog isolates in the Hunan province. Many studies have demonstrated that RABV isolates from different hosts have specific genetic sequences and they are strongly clustered according to geographical origin (Bourhy et al., 1999; Coetzee et al., 2008; Tohma et al., 2014). In China, the human RABV usually shows a close relationship with the dog virus from the same area (Tao et al., 2009). So, we speculated that bitten by a rabies dog was the most probably infectious pathway in this case. We also noticed that the *G* gene of CCS1501H was most closely related to a dog isolates in Chongqing, a neighboring province of Hunan. However, the gene flow of RABV principally occurring amongst geographically adjacent countries had been demonstrated previously (Guo et al., 2013).

In China, the Hunan province is a rabies high incidence region with 3,634 recorded human cases up to 2010 (Zhang et al., 2005; Gao et al., 2011). Surveillance data revealed that there were 16 human cases in Yuanjiang City between 2010 and 2015, including 4 cases in the donor's village. Most RABV isolates in the Hunan province belong to the China I and II lineages, with the dog being the main reservoir host, no wildlife animals have found in the public database till now (Guo et al., 2013; Tao et al., 2013). Detailed interviews with family members revealed that the two kidney recipients had no animal exposure history and that the organ donor had an unexplained wound on his forehead 2 months before symptom onset. The donor was just a 22-month-old boy, the abilities of communication and avoiding potential dangers were limited. Many stray dogs were roaming in the donor's village. Therefore, we speculated that the donor's forehead wound might have been caused by a dog bite, which could have been the source of the initial infection.

The incubation period of RABV can vary from 5 days to several years but is usually 2 or 3 months (Boland et al., 2014). The typical RABV incubation period is believed to be shortened in transplant patients, especially when they are in an immunosuppressive state (Srinivasan et al., 2005; Maier et al., 2010). The average incubation period in the kidney recipients in the present study was 42 days, which was similar to that reported previously in Beijing. The kidney recipients' conditions deteriorated quickly, and both died 3 or 4 days after the onset of symptoms. The liver recipient was readmitted to hospital 33 days after transplantation and died of respiratory failure 4 days later. Though there were no symptoms related to rabies and no specimens to confirm this, we suspect that the liver recipient might also have been infected with RABV.

In actually, rabies was included in the differential diagnosis of encephalitis for the organ donor, and the viral-specific antibodies in the serum were tested, but the result was negative. In addition, the lack of animal exposure history and the short window of time for organ transplantation possibly contributed to the misdiagnosis. Serum and cerebrospinal fluid antibody testing are of limited value because seroconversion occurs late in the course of the disease (Schuller et al., 1979); RABV antibody was detected in only 20% of unvaccinated rabies patients tested within 1–26 days of disease onset (Hemachudha, 1994; Hemachudha et al., 2002). Other molecular methods, such as real time RT-PCR, have proven valuable for the detection of RABV, especially for antemortem diagnoses (Mani et al., 2014; Dupuis et al., 2015). Multiple detection techniques and multiple tissue samples (e.g., saliva, cerebrospinal fluid, serum, skin, and concentrated urine) would be beneficial for the diagnosis and differentiation of rabies.

In summary, we reported a suspected case of rabies transmission via organ transplantation. Although we could not confirm the donor's rabies infection due to the lack of clinical specimens, both kidney recipients had post-transplant symptoms of rabies during the same period, and RABV nucleic acid was found in their saliva samples. In addition, the epidemiological investigation and molecular results supported the conclusion. Based on this case, if patients have unexplained meningoencephalitis, rabies should be excluded using a combination of multiple techniques, especially in potential organ donors. Although the risk of donor-derived disease is inherent in the process of organ transplantation and cannot be eliminated, raising awareness of the risk is the best way to reduce the occurrence of these transmissions, especially to clinicians, who play a key role in the potential donor screening. A detail animal contacts and travel history will be very helpful to the differential diagnosis. Furthermore, education can help to prevent the recurrence of similar tragedy, especially in rabies epidemic regions.

## Author contributions

HZ, SC, and ML collected epidemiological data. XO and DY performed experimental detection. RSZ, DY, and JC performed RT-PCR and sequencing. GL, TJ, and PL performed next generation sequencing. JC, GL, TJ, BS, and RLZ designed study and analyzed data. JC, GL, TJ, FY, and DH wrote the first draft of the manuscript. BS and RLZ revised the manuscript.

### Conflict of interest statement

The authors declare that the research was conducted in the absence of any commercial or financial relationships that could be construed as a potential conflict of interest.
