# Supplementary material for: Epidemiological and Genetic Characteristics of Rabies Virus Transmitted Through Organ Transplantation
Source: Front Cell Infect Microbiol. 2018 Mar 27;8:86. doi: 10.3389/fcimb.2018.00086 (PMC5880885; doi:10.3389/fcimb.2018.00086)
Supplement: Supplementary file 1 [file Table1.DOCX]

**Supplementary Table S1** List of viruses used in the full-length genome analyses.

| **Country** | **Virus Name** | **Source** | **Year** | **Phylogenetic clade - subclade** | **Accession number** |
| --- | --- | --- | --- | --- | --- |
| Senegal | 93005SEN | Dog | 1992 | Africa-2 | KX148239 |
| Nigeria | DRV_NG11 | Dog | 2011 | Africa-2 | KC196743 |
| Niger | 90010NIG | Dog | 1990 | Africa-2 | KX148231 |
| Mauritania | 93011MAU | Dog | 1993 | Africa-2 | KX148236 |
| Ivory Coast | 01007CI | Dog | 2001 | Africa-2 | KX148235 |
| Guinea | 90024GUI | Dog | 1990 | Africa-2 | KX148244 |
| Chad | 96009TCH | Dog | 1996 | Africa-2 | KX148241 |
| Central African Republic | CAR_11_001 | Human | 2011 | Africa-2 | KF977826 |
| Burkina Faso | 95047HAV | Dog | 1995 | Africa-2 | KX148230 |
| Benin | 86097BEN | Cat | 1986 | Africa-2 | KX148107 |
| South Africa | 15001AFS | Yellow mongoose | 2013 | Africa-3 | KX148220 |
| South Africa | 15003AFS | Ground squirrel | 2014 | Africa-3 | KX148221 |
| South Africa | 14018AFS | Feline | 2000 | Africa-3 | KX148223 |
| Botswana | 14016BOT | Wildcat | 2009 | Africa-3 | KX148218 |
| Greenland | Gra 02.09-7-S | Sheep | 2009 | Arctic-related | LM645040 |
| Greenland | Gra 01.10-7-F | Polar fox | 2010 | Arctic-related | LM645043 |
| USA | 91005USA | Red fox | 1990 | Arctic-related | KX148106 |
| South Korea | BV9901PJ | Raccoon dog | 1999 | Arctic-related | KC171645 |
| Russia | 1410KOM | Deer | 2008 | Arctic-related | JQ944707 |
| Nepal | 99001NEP | Dog | 1998 | Arctic-related | KX148228 |
| Pakistan | Pk_23 | Cow | 2010 | Arctic-related | HE802675 |
| China | CQH1202D | Dog | 2012 | Arctic-related | KM272192 |
| Greenland | 86083GRO | Dog | 1980 | Arctic-related | KX148105 |
| Afghanistan | 04035AFG | Dog | 2002 | Arctic-related | KX148226 |
| Afghanistan | 02052AFG | Dog | 2002 | Arctic-related | KX148225 |
| India | NNV_RAB_H | Human | 2006 | Arctic-related | EF437215 |
| India | Serotype_1 | Human | 2004 | Arctic-related | AY956319 |
| Argentina | 97004ARG | Bat | 1997 | Bat | KX148269 |
| Brazil | BR_DR1 | Bat | 2000 | Bat | AB519642 |
| French Guiana | 09035FRA | Bat | 2009 | Bat | KX148100 |
| Mexico | Coati_3639 | Coati | 2009 | Bat | JQ685963 |
| Mexico | MEXSK3644 | Spotted skunk | 2009 | Bat | JQ685929 |
| USA | A093504 | Bat | 2009 | Bat | JQ685950 |
| USA | AZ3003 | Bat | 2009 | Bat | JQ685971 |
| USA | TN209 | Bat | 2005 | Bat | JQ685902 |
| India | 97002IND | Human | 1997 | Indian Subcontinent | KX148246 |
| Nepal | 11001NEP | Cattle | 2011 | Indian Subcontinent | KX148108 |
| Sri Lanka | RV2417 | Dog | 2008 | Indian Subcontinent | KF154999 |
| Sri Lanka | H_1413_09 | Golden palm civet | 2009 | Indian Subcontinent | AB635373 |
| Nepal | 09029NEP | Buffalo | 2009 | Indian Subcontinent | KX148245 |
| China Taiwan | R2012_26 | Ferret badger | 2012 | Asian | KF620487 |
| China Taiwan | R2013_01 | Ferret badger | 2013 | Asian | KF620489 |
| Vietnam | 01016VNM | Dog | 2001 | Asian | KX148254 |
| Philippines | 94281PHI | Dog | 1994 | Asian | KX148261 |
| Philippines | 04030PHI | Dog | 2004 | Asian | KX148260 |
| Laos | 02003LAO | Dog | 2002 | Asian | KX148258 |
| Cambodia | 99016CBG | Dog | 1999 | Asian | KX148250 |
| Cambodia | 99011CBG | Dog | 1998 | Asian | KX148251 |
| China | 02050CHI | Human | 1992 | Asian | KX148267 |
| China | CQ92 | Dog | 1992 | Asian | GU345746 |
| China | CYN1009D | Dog | 2010 | Asian | JQ730682 |
| China | RV_J | Human | 1986 | Asian | GU345747 |
| Indonesia | 03003INDO | Dog | 2003 | Asian | KX148266 |
| Laos | 99010LAO | Dog | 1999 | Asian | KX148255 |
| Myanmar | 99009BIR | Dog | 1999 | Asian | KX148247 |
| Thailand | 8764THA | Human | 1983 | Asian | EU293111 |
| China | CNM1103C | Cow | 2011 | Cosmopolitan | KC252633 |
| China | DRV | Deer | 1989 | Cosmopolitan | DQ875051 |
| China | WQ14_RF | Red fox | 2014 | Cosmopolitan | KM016899 |
| USA | 1088 | Woodchuck | 2008 | Cosmopolitan | AB645847 |
| Iran | 96299IRA | Wolf | 1996 | Cosmopolitan | KX148185 |
| Turkey | 94009TUR | Dog | 1993 | Cosmopolitan | KX148165 |
| Tanzania | RV2772 | Dog | 2009 | Cosmopolitan | KF155002 |
| Somalia | 93006SOM | Jackal | 1993 | Cosmopolitan | KX148199 |
| Slovenia | 94099SLN | Red fox | 1994 | Cosmopolitan | KX148144 |
| Slovenia | 94094SLN | Red fox | 1994 | Cosmopolitan | KX148132 |
| Saudi Arabia | 04034ARS | Fox | 2004 | Cosmopolitan | KX148176 |
| Russia | 8054f | Red fox | 2011 | Cosmopolitan | KC595282 |
| Russia | 1352KRA | Dog | 2008 | Cosmopolitan | JQ944706 |
| Poland | 96042POL | Red fox | 1996 | Cosmopolitan | KX148151 |
| Mexico | MEXSK3636 | Spotted skunk | 2009 | Cosmopolitan | JQ685975 |
| Morocco | 08342MAR | Dog | 2008 | Cosmopolitan | KX148193 |
| Montenegro | 86058YOU | Cow | 1978 | Cosmopolitan | KX148104 |
| Madagascar | 04033MAD | Dog | 2004 | Cosmopolitan | KX148209 |
| Kenya | 14015ITA | Human | 2014 | Cosmopolitan | KX148207 |
| Israel | 96312ISR | Dog | 1996 | Cosmopolitan | KX148181 |
| Iran | 93019IRA | Jackal | 1993 | Cosmopolitan | KX148212 |
| Hungary | 93089HON | Red fox | 1993 | Cosmopolitan | KX148137 |
| Ethiopia | RV2985 | Ethiopian wolf | 2014 | Cosmopolitan | KP723638 |
| Brazil | BR_Pfx1 | Brazilian wild fox | 2002 | Cosmopolitan | AB362483 |
| China | 98011CHI | Dog | 1998 | Asian- ChinaI | KX148265 |
| China | JX09_17 | Ferret badger | 2009 | Asian- ChinaI | KC762941 |
| China | CNM1104D | Dog | 2011 | Asian- ChinaI | KC252634 |
| China | CSX0904D | Dog | 2009 | Asian- ChinaI | JQ970487 |
| China | FJ009 | Dog | 2008 | Asian- ChinaI | FJ866836 |
| China | SH06 | Dog | 2006 | Asian- ChinaI | GU345748 |
| China | Shaanxi-HZ-6 | Dog | 2009 | Asian- ChinaI | KC977995 |
| China | FJDRV | Dog | 2008 | Asian- ChinaI | JN609295 |
| China | CSD0708D | Dog | 2007 | Asian- ChinaI | JQ970486 |
| China | CJX0906D | Dog | 2009 | Asian- ChinaI | JQ970485 |
| China | CJS0621D | Dog | 2006 | Asian- ChinaI | JQ970481 |
| China | SXYL15 | Cattle | 2015 | Asian- ChinaI | KR230090 |
| China | BJ2011E | Horse | 2011 | Asian- ChinaI | JQ423952 |
| China | IMDRV_13 | Fallow deer | 2013 | Asian- ChinaI | KJ564280 |
| China | JZ13_Lv | Human | 2013 | Asian- ChinaI | KJ004416 |
| China | D01 | Dog | 2008 | Asian- ChinaI | FJ712193 |
| China | WH11 | Donkey | 2011 | Asian- ChinaI | JQ647510 |
| China | JX10_37 | Ferret badger | 2010 | Asian- ChinaI | KF726853 |
| China | CJX0903D | Dog | 2009 | Asian- ChinaI | JQ970484 |
| China | CJS0636D | Dog | 2006 | Asian- ChinaI | JQ970482 |
| China | FJ008 | Dog | 2008 | Asian- ChinaI | FJ866835 |
| China | DRV_AH08 | Dog | 2008 | Asian-ChinaI | HQ450385 |
| China | 02046CHI | Dog | 1994 | Asian-ChinaII | KX148264 |
| China | JX09_18 | Ferret badger | 2009 | Asian-ChinaII | KF726852 |
| China | CHN0802D | Dog | 2008 | Asian-ChinaII | JQ970480 |
| China | GD_SH_01 | Pig | 2011 | Asian-ChinaII | JX088694 |
| China | HN10 | Human | 2006 | Asian-ChinaII | EU643590 |
| China | GX4 | Dog | 1994 | Asian-ChinaII | GU358653 |
| China | JX08_45 | Ferret badger | 2008 | Asian-ChinaII | GU647092 |
| China | F02 | Ferret badger | 2008 | Asian-ChinaII | FJ712195 |
| China | F04 | Ferret badger | 2008 | Asian-ChinaII | FJ712196 |
| USA | SADB19 | - | - | Vaccine strain | M31046 |
| China | CTN-1 | - | - | Vaccine strain | FJ959397 |
| China | SRV9 | - | - | Vaccine strain | AF499686 |
| France | PV | - | - | Vaccine strain | M13215 |
