# Supplementary material for: Epidemiological and Genetic Characteristics of Rabies Virus Transmitted Through Organ Transplantation
Source: Front Cell Infect Microbiol. 2018 Mar 27;8:86. doi: 10.3389/fcimb.2018.00086 (PMC5880885; doi:10.3389/fcimb.2018.00086)
Supplement: Supplementary file 2 [file Table2.DOCX]

**Supplementary Table S2** List of Chinese isolates used in the full-length N and G genes analysis.

| **Lineages** | **Accession number（G）** | **Accession number（N）** | **Strain** | **Region** | **Host** | **Year** |
| --- | --- | --- | --- | --- | --- | --- |
| China I | FJ418879 | EU159390 | FS | Guangxi | Dog | 1998 |
| China I | GQ857471 | EU159401 | WJ | Zhejiang | Dog | 2008 |
| China I | GQ857472 | EU159400 | H89 | Anhui | Dog | 1989 |
| China I | JF819598 | JF819624 | CYN1007H | Yunnan | Human | 2010 |
| China I | JF819600 | JF819621 | CYN1005D | Yunnan | Dog | 2010 |
| China I | JN936757 | JN974861 | CSC1013D | Sichuan | Dog | 2010 |
| China I | JN936708 | JN974840 | CHN0602D | Hunan | Dog | 2006 |
| China I | DQ849054 | EU159372 | FY12 | Anhui | Dog | 2005 |
| China I | EU267757 | DQ666302 | Sq21 | Henan | Dog | 2005 |
| China I | JN936714 | JN974844 | CHN0701D | Hunan | Dog | 2007 |
| China I | JN936717 | JN974846 | CHN0809D | Hunan | Dog | 2008 |
| China I | JN936709 | JN974841 | CHN0610H^a^ | Hunan | Human | 2006 |
| China I | JN936712 | JN974843 | CHN0635H^a^ | Hunan | Human | 2006 |
| China I | EU267758 | DQ666304 | Henan Sq35 | Henan | Dog | 2005 |
| China I | DQ849059 | DQ666316 | WG432 | Hunan | Dog | 2005 |
| China I | JN936746 | JN974855 | CJX0903D | Jiangxi | Dog | 2009 |
| China I | GU233763 | GU233765 | JX09-17(fb) | Jiangxi | Ferret badger | 2009 |
| China I | GQ472553 | GQ472472 | GXNN2 | Guangxi | Dog | 2007 |
| China I | FJ418885 | HQ118102 | gk5 | Guizhou | Dog | 2006 |
| China I | JN936704 | JN974833 | CGZ1030D | Guizhou | Dog | 2010 |
| China I | JN936744 | JN974853 | CJX0901D | Jiangxi | Dog | 2009 |
| China I | DQ849061 | EU159380 | WH5 | Hubei | Dog | 2005 |
| China I | DQ849063 | EU159377 | QC | Hubei | Human | 2006 |
| China I | JN936705 | JN974836 | CHN0503D | Hunan | Dog | 2005 |
| China I | EU267754 | DQ666298 | Sq6 | Henan | Dog | 2005 |
| China I | EU267771 | DQ666319 | Hunan Xx35 | Hunan | Dog | 2004 |
| China I | JN936732 | JN974849 | CJS0840H | Jiangsu | Human | 2008 |
| China I | JN936733 | JN974850 | CJS0841D | Jiangsu | Dog | 2008 |
| China I | JN936745 | JN974854 | CJX0902D^a^ | Jiangxi | Dog | 2009 |
| China I | JN936760 | JN974863 | CSC1016D | Sichuan | Dog | 2010 |
| China I | JN936740 | JQ970483 | CJS0848D | Jiangsu | Dog | 2008 |
| China I | JN936777 | JN974869 | CSD0935D | Shandong | Dog | 2009 |
| China I | EF556198 | EF556197 | Wz0(H) | Zhejiang | Human | 2006 |
| China I | GQ857470 | EU159397 | LH | Zhejiang | Dog | 2006 |
| China I | EU700030 | EU700032 | Wz1(H) | Zhejiang | Human | 2008 |
| China I | JN936792 | JN974878 | CZJ0814D | Zhejiang | Dog | 2008 |
| China I | EU700029 | EU700031 | Hu1 | Beijing | Human | 2007 |
| China I | JQ699241 | JN974876 | CSX0903D | Shaanxi | Dog | 2009 |
| China I | FJ418883 | EU159392 | SBH | Shanghai | Human | 1992 |
| China I | FJ418884 | EU159393 | SBD | Shanghai | Dog | 1992 |
| China I | FJ418886 | EU159394 | SH06 | Shanghai | Dog | 2006 |
| China I | JN936723 | JN974848 | CHN0903D | Hunan | Dog | 2009 |
| China I | KP072050 | KP072029 | CYN1382D | Yunnan | Dog | 2013 |
| China I | KT221138 | KT894584 | SXDTD13 | Shanxi | Dog | 2013 |
| China I | KF663544 | KF663528 | ShaanxiRab018 | Shaanxi | Human | 2012 |
| China I | KF663545 | KF663529 | ShaanxiRab020 | Shaanxi | Dog | 2012 |
| China I | KT221139 | KT894580 | SHPDD02 | shanghai | Dog | 2013 |
| China I | KP202413 | KP202440 | CYN14107H | Yunnan | Human | 2014 |
| China I | KP202414 | KP202441 | CYN14108D | Yunnan | Dog | 2014 |
| China I | KU198997 | KU198994 | SXDON15 | Shanxi | donkey | 2015 |
| China I | JQ699258 | KC465379 | CNX1101H | Ningxia | Human | 2011 |
| China I | KJ564280 | KJ564280 | IMDRV-13 | Inner Mongolia | Deer | 2013 |
| China I | JF819601 | JF819619 | CYN1003C | Yunnan | Cattle | 2010 |
| China I | KT221119 | KT221096 | CQQJD07 | Chongqing | Dog | 2007 |
| China I | FJ602453 | - | LuoH | Henan | Human | 2007 |
| China I | JN936763 | - | CSC1019H | Sichuan | Human | 2010 |
| China I | GU186382 | - | HuNDB07 | Hunan | Dog | 2007 |
| China I | GU186388 | - | CQFJ02 | Chongqing | Dog | 2007 |
| China I | JN936706 | - | CHN0505D | Hunan | Dog | 2005 |
| China I | GU186407 | - | SXAZ02 | Shanxi | Dog | 2007 |
| China I | GU186408 | - | SXLF03 | Shanxi | Dog | 2008 |
| China I | GU186384 | - | HuNDB28 | Hunan | Dog | 2005 |
| China I | JN936702 | - | CGZ1026H | Guizhou | Human | 2010 |
| China I | EU267760 | - | Wh20 | Hubei | Dog | 2006 |
| China I | FJ602454 | - | WHqs | Hubei | Dog | 2007 |
| China I | FJ602451 | - | HNC | Hubei | Cattle | 2007 |
| China I | JN936691 | - | CGX0603D | Guangxi | Dog | 2006 |
| China I | GU186385 | - | HuNDB33 | Hunan | Dog | 2006 |
| China I | JN936779 | - | CSD1046H | Shandong | Human | 2010 |
| China I | FJ602447 | - | CQH | Chongqing | Human | 2007 |
| China I | GU186389 | - | CQJLP01 | Chongqing | Dog | 2008 |
| China I | GU186405 | - | SDJN02 | Shandong | Cattle | 2007 |
| China I | GU186403 | - | SDDZ01 | Shandong | Cattle | 2006 |
| China I | FJ825133 | - | F01 | Zhejiang | Ferret badger | 2008 |
| China I | KT221140 | - | GSHSD14 | Gansu | Dog | 2014 |
| China I | KT221120 | - | CQBND01 | Chongqing | Dog | 2007 |
| China I | GU186381 | - | HuNDB06 | Hunan | Dog | 2006 |
| China I | GU186383 | - | HuNDB16 | Hunan | Dog | 2005 |
| China I | - | KU928250 | NX15 | Ningxia | camel | 2015 |
| China I | - | JN974832 | CGZ0924H | Guizhou | Human | 2009 |
| China I | - | EF611081 | hubei070308 | Hubei | Cattle | 2004 |
| China I | - | KT894574 | TJC14 | Tianjing | cow | 2014 |
| China II | JN936788 | JN974877 | CZJ0803F | Zhejiang | Ferret badger | 2008 |
| China II | FJ719749 | FJ719751 | JX08-47 | Jiangxi | Ferret badger | 2008 |
| China II | FJ719752 | FJ719753 | JX08-48 | Jiangxi | Ferret badger | 2008 |
| China II | FJ719756 | FJ598135 | ZJ-LA | Zhejiang | Ferret badger | 2008 |
| China II | JN936688 | JN974823 | CGD0801D | Guangdong | Dog | 2008 |
| China II | GQ472547 | GQ472468 | GXHXB | Guangxi | Dog | 2007 |
| China II | JN936711 | JN974842 | CHN0633D | Hunan | Dog | 2006 |
| China II | GQ472557 | GQ472477 | GXQZD | Guangxi | Dog | 2006 |
| China II | JN936720 | JN974847 | CHN0813H | Hunan | Human | 2008 |
| China II | GQ472558 | - | GXSL | Guangxi | Cattle | 2005 |
| China II | GU186380 | - | HuNDB03 | Hunan | Dog | 2006 |
| China II | GU186386 | - | HuNPB01 | Hunan | Pig | 2006 |
| China II | JN936719 | - | CHN0812D | Hunan | Dog | 2008 |
| China II | FJ602452 | - | LU | Henan | Deer | 1993 |
| China II | GQ472559 | - | GXWX | Guangxi | Pig | 2005 |
| China II | - | KT894573 | HNNYD03 | Hunan | Dog | 2011 |
| China II | - | EU086184 | 02049CHI | China | Sika Deer | 1993 |
| China III | KJ152775 | KJ152773 | XJTCS01 | Xinjiang | sheep | 2013 |
| China III | DQ875050 | DQ875050 | MRV | Henan | Mouse | 1989 |
| China III | DQ875051 | DQ875051 | DRV | Jilin | Deer | 1989 |
| China III | KM016899 | KM016899 | WQ14-RF | Inner Mongolia | Red fox | 2014 |
| China III | EU267772 | DQ666320 | Wx0(H) | Jiangsu | Human | 2004 |
| China III | EU267745 | - | Al0(H) | Guizhou | Human | 2004 |
| China III | - | KU041696 | XJTKSFOX14 | Xinjiang | Fox | 2014 |
| China III | - | KJ748635 | NMC03 | Inner Mongolia | cattle | 2014 |
| China III | - | KJ748633 | NMFOX01 | Inner Mongolia | fox | 2014 |
| China III | - | KJ748636 | NMC04 | Inner Mongolia | cattle | 2014 |
| China III | - | KJ748632 | NMCAM02 | Inner Mongolia | camel | 2014 |
| China IV | EU284095 | EU284093 | NeiMeng927A | Inner Mongolia | Raccoon dog | 2007 |
| China IV | EU284096 | EU652444 | NeiMeng927B | Inner Mongolia | Raccoon dog | 2007 |
| China IV | EU284097 | EU652445 | NeiMeng1025B | Inner Mongolia | Raccoon dog | 2007 |
| China IV | KM272192 | KM272192 | CQH1202D | Qinghai | Dog | 2012 |
| China V | FJ418876 | EU159387 | J | Ningxia | Human | 1985 |
| China V | DQ849072 | EU159388 | CQ92 | Chongqing | Dog | 1992 |
| China V | AY009098 | - | CNX8511 | Ningxia | Human | 1985 |
| China V | AY009099 | - | CNX8601 | Ningxia | Human | 1986 |
| China V | GU186397 | - | CQWS01 | Chongqing | Dog | 2005 |
| China VI | JQ730682 | JX276405 | CYN1009D | Yunnan | Dog | 2010 |
| China VI | EU275242 | EU275243 | Tc06 | Yunnan | Dog | 2006 |
| China VI | DQ849069 | FJ594278 | N11 | Guangxi | Dog | 1997 |
| China VI | GQ472552 | DQ866111 | GXN119 | Guangxi | Dog | 2000 |

a: The lineages was referred to the G gene and the N genes of these strains clustered into China II lineage.
